# Whole-head recording of chemosensory activity in the marine annelid *Platynereis dumerilii*

**DOI:** 10.1098/rsob.180139

**Published:** 2018-10-31

**Authors:** Thomas F. Chartier, Joran Deschamps, Wiebke Dürichen, Gáspár Jékely, Detlev Arendt

**Affiliations:** 1 Developmental Biology Unit, European Molecular Biology Laboratory, Meyerhofstraße 1, 69117 Heidelberg, Germany; 2 Cell Biology and Biophysics Unit, European Molecular Biology Laboratory, Meyerhofstraße 1, 69117 Heidelberg, Germany; 3 Living Systems Institute, University of Exeter, Stocker Road, Exeter EX4 4QD, UK

**Keywords:** *Platynereis*, marine chemosensation, calcium imaging, microfluidics, neuronal activity, invertebrates

## Abstract

Chemical detection is key to various behaviours in both marine and terrestrial animals. Marine species, though highly diverse, have been underrepresented so far in studies on chemosensory systems, and our knowledge mostly concerns the detection of airborne cues. A broader comparative approach is therefore desirable. Marine annelid worms with their rich behavioural repertoire represent attractive models for chemosensation. Here, we study the marine worm *Platynereis dumerilii* to provide the first comprehensive investigation of head chemosensory organ physiology in an annelid. By combining microfluidics and calcium imaging, we record neuronal activity in the entire head of early juveniles upon chemical stimulation. We find that *Platynereis* uses four types of organs to detect stimuli such as alcohols, esters, amino acids and sugars. Antennae are the main chemosensory organs, compared to the more differentially responding nuchal organs or palps. We report chemically evoked activity in possible downstream brain regions including the mushroom bodies (MBs), which are anatomically and molecularly similar to insect MBs. We conclude that chemosensation is a major sensory modality for marine annelids and propose early *Platynereis* juveniles as a model to study annelid chemosensory systems.

## 1. Introduction

Chemical signals are central to animal behaviour, including feeding, predation, courtship and mating, aggregation, defence, habitat selection and communication [1]. Adapting to variable habitats and changing chemical landscapes, animals have evolved a broad variety of chemosensory organs. Investigations of chemosensory systems in mammals, insects and nematodes have provided insights into the molecular and cellular basis of how chemical information is encoded into neuronal activity [2–4]. While similar circuit architectures can be found in distant species at some steps of information processing, this appears to be no general rule [5,6]. Genomic studies have revealed that receptor proteins are highly diverse in the animal kingdom [7] and can be entirely different between distant species—vertebrates and insects, for example, use distinct types of receptors [8,9]. Hence, a broader comparative approach will facilitate the elucidation of both general operating principles and evolutionary origins of animal chemosensation. Notwithstanding studies in fish and crustaceans [10,11], and to a lesser extent in molluscs [12,13], our current understanding of animal chemosensation still mainly concerns terrestrial and airborne cues. Marine animals thus deserve more attention.

Marine annelids, traditionally referred to as ‘polychaetes’, represent an attractive group for chemosensory studies. These worms, represented by more than 10 000 species, are typically free-living, burrow in the marine sediment or build tubes. They are known to respond to chemical signals in reproduction, feeding, aggression, avoidance, aggregation, environment probing, larval settlement and metamorphosis [14]. Marine annelids are suited for electrophysiological [15–18] as well as behavioural [19–22] studies, and their nervous system is anatomically and histologically well described [23–31]. Potential chemoreceptor proteins have been identified in the first published annelid genomes, which contain homologues for insect receptors (41 ionotropic receptors (IRs) and 12 gustatory receptor-like receptors (GRs)), but apparently not for mammalian ones (olfactory receptors (ORs)) [32,33]. Despite these advantages, the physiology of chemical sensing in annelids is scarcely known (for an up-to-date review on annelid chemosensation, see [34, ch. II]).

Unlike terrestrial annelids such as leeches and earthworms, marine annelids possess elaborate head sensory organs with diverse morphologies [35]. Nuchal organs, paired ciliated cavities located at the back of the head, are considered an annelid synapomorphy [36] and are generally regarded as chemosensory. Based on cell morphology, they are the best candidate chemosensory organs, though no physiological support yet substantiates this claim. Palpae, or palps, the most important head appendages for phylogenetic systematization, have been proposed to be chemosensory based on cell ultrastructure and activity-dependent cell labelling [31,37,38]. Similar claims based on ultrastructure were made for antennae and tentacular cirri, the two other major types of head appendages [31,39]. Gross in 1921 [19] showed that the removal of palps or antennae, and to a lesser extent of tentacular cirri, lengthens the reaction time of the nereidid *Nereis virens* to ionic solutions. Yet, physiological evidence is scarce, and at present no direct experimental proof of chemosensitivity exists for any of these four head organs.

**Figure 1.**
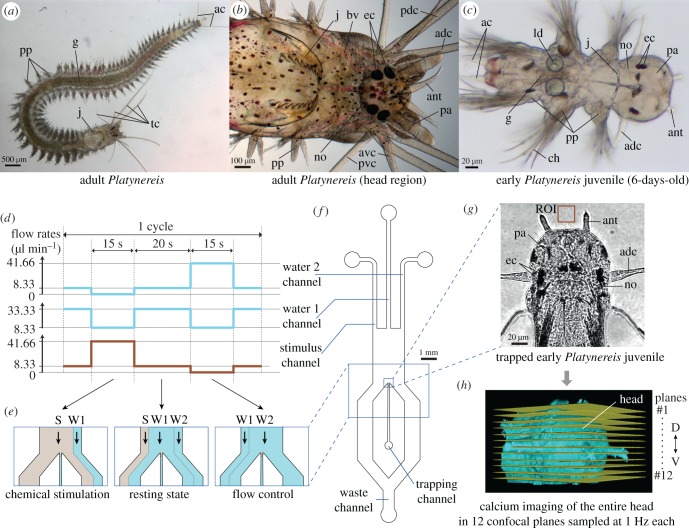
*Platynereis* and the microfluidic device for precise chemical stimulations. (*a*–*c*) Light microscopy pictures of *Platynereis* at the adult (*a*,*b*) and early juvenile (*c*) stages, showing antennae (ant), palps (pa) and tentacular cirri (tc, adc, avc, pdc, pvc). Further abbreviations: see the abbreviations list. (*a*–*c*) Antje Fischer ^©^. (*d*) Channel flow rates used to generate (*e*) the different flow patterns. The sum of the three channels' flow rates is constant. (*f*) Schematic of the microfluidic device. The three inlet channels are operated by computer-controlled pumps. Single animals are introduced manually in the trapping channel and immobilized at its end. (*g*) An immobilized early juvenile, with its head freely exposed to the seawater flows. Confocal image with transmitted light illumination. (*h*) Calcium signals upon chemical stimulation are recorded with a confocal microscope in 12 optical planes sampling the whole head volume.

We report here a comprehensive study of head chemosensory organ physiology in the marine annelid *Platynereis dumerilii* (figure 1*a–c*), which can be easily kept in the laboratory and is amenable to molecular studies. This species belongs to nereidids, the family regarded as best representing the annelid nervous system [40, p. 735]. Rather than adults, we chose to study early juveniles around the 6-days-post-fertilization (6 dpf) stage, which are already equipped with nuchal organs, palps, antennae and one pair of tentacular cirri (figure 1*c*). They feed, crawl, swim and display various natural behaviours such as phototaxis, startling, escaping or silk spinning. They present experimental advantages comparable to those of the nematode *Caenorhabditis elegans*, being transparent, developmentally synchronous, easily obtainable in high numbers and suitable for whole-body light and electron microscopy. A whole-body atlas of gene expression available for this developmental stage [41] constitutes a unique resource which facilitates the characterization of cell types. Moreover, a connectomic resource exists at a larval stage that has proved powerful in the reconstruction of whole-body neuronal circuits, notably in the context of phototaxis and ciliated locomotion [42–44].

Four stimuli (1-butanol, amyl acetate, glutamate and sucrose) were chosen for their different physico-chemical properties and their likely ecological relevance to *Platynereis*. Short alcohols trigger behavioural reactions in *Nereis* [45]. Amyl acetate can act as a conditioned stimulus for associative learning in the aquatic snail *Lymnea* [46]. Glutamate elicits behavioural responses in *Nereis* [45], and amino acids, in general, are relevant aquatic chemical cues for various marine animals [12,38,47–50]. Sugars can be degradation products of the polysaccharides contained in plants such as eelgrass and seagrass, or in algae, on which nereidid polychaetes are known to feed [19,51]. However, except for a pH and salinity preferendum [52], nothing is known regarding chemoreception and relevant chemical cues in early *Platynereis* juveniles.

Using a customized microfluidic device for animal immobilization and precise stimulus delivery, we performed whole-head functional imaging in early juveniles ubiquitously expressing the genetically encoded calcium sensor GCaMP6s. We found that nuchal organs, palps, antennae and tentacular cirri are chemosensory, though with different degrees of specialization: for example, antennae responded to all stimulants, while nuchal organs were most sensitive to amyl acetate and sucrose, but did not respond to glutamate. We observed a chemically evoked activity in other regions including the mushroom bodies (MBs), which could potentially be involved in learning phenomena. We also described a prominent oscillatory activity in the larval apical organ, however not obviously linked to chemical stimulations. We provide the first direct evidence of chemosensory function in annelid head organs and lay the ground for future investigations of sensory integration.

## 2. Results

### 2.1. Establishment of a functional imaging assay system for chemosensation in early *Platynereis* juveniles

We designed a simple microfluidic device, made of the transparent polymer polydimethylsiloxane (PDMS) and fabricated by soft lithography. The device is symmetric, has a uniform height of 60 µm and consists of a single chamber in which a constant flow of natural seawater is established. Three inlet channels generate three parallel, non-mixing water streams thanks to a laminar flow regime. Changing the relative flow rates between channels allows to expose the chamber's centre to any of the streams (figure 1*d*,*e*). With one side stream being used to deliver a chemical stimulus and the other to deliver the solvent alone (seawater), a flow control is performed in each experiment that allows to separate the purely chemical sensory input from the mechanical one. An early *Platynereis* juvenile can be immobilized at the end of a central trapping channel (figure 1*f*), where its head is exposed to the water flows (figure 1*g*; see electronic supplementary material, figure S1A,B for a detailed description of the set-up).

We performed calibration experiments with a dye to quantify the actual changes of stimulant concentration at the level of the animal's head (see electronic supplementary material, figure S1C and Material and methods). With this set-up, a change from zero to maximum stimulant concentration (stimulus onset) can be completed within 0.9 ± 0.3 s, with a delay of 3.2 ± 0.5 s compared with the pump triggering time. The reverse change (stimulus offset) can be completed within 1.0 ± 0.2 s, with a delay of 3.0 ± 0.5 s. Slight timing differences between these two events stem from the geometrical asymmetry between central and lateral streams.

To survey calcium activity in the entire head, we immobilized juveniles ubiquitously expressing GCaMP6s and acquired images with a confocal microscope from 12 horizontal, equally spaced optical sections sampling the whole head volume (figure 1*h*). With the whole stack being acquired within a second, a 1 Hz temporal resolution was obtained for each of the 12 planes; hence, the precision of stimulus delivery was deemed satisfactory.

### 2.2. Ten distinct sets of cells show activity during chemical stimulation experiments

To comprehensively identify head regions active in the context of chemical stimulations, we imaged calcium activity across the entire head in response to four stimulants (see Introduction). For each stimulant, three to four experiments were conducted for each of nine animals (only eight for 1-butanol). A single experiment consisted of three identical cycles: each cycle comprised a chemical stimulus and a flow control, lasting 15 s each and spaced by 20 s resting intervals (figure 1*d*).

**Figure 2.**
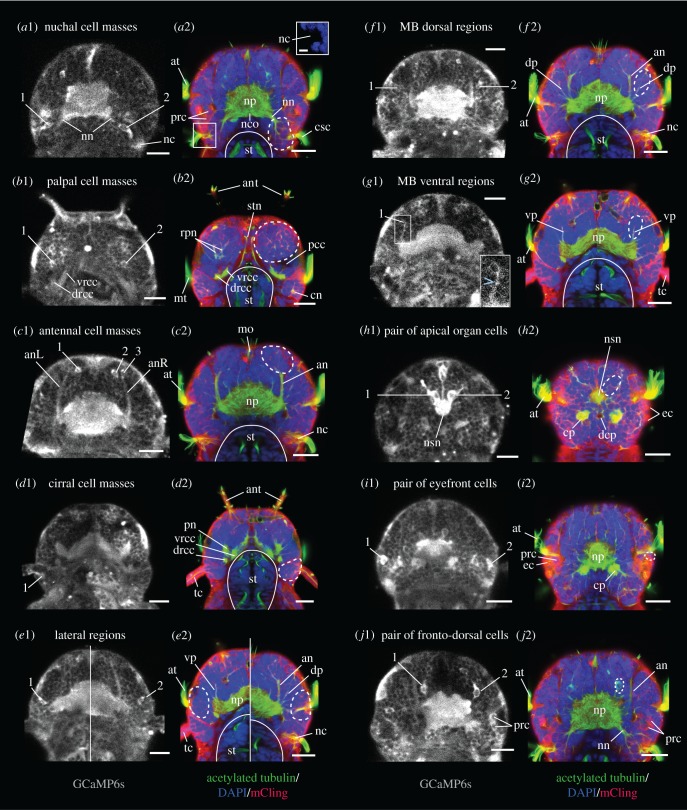
Calcium images and anatomical stainings in dorsal view, showing the head regions active in the context of chemical stimulations. (*a*1, *b*1, … *j*1) GCaMP6s signal from single confocal planes, averaged over several consecutive time points. Individual active cells are pointed at by numbers. (*a*2, *b*2, … *j*2) Acetylated tubulin immunostaining (green), counterstained for nuclear DNA (DAPI, blue) and membranes (mCling, red), averaged over several consecutive confocal planes. The approximate boundaries of the active regions are indicated by white dashed lines, and the outlines of the stomodeum by white solid lines. The dorsoventral position of the planes, as well as lateral views of the anatomical stainings, are shown in the electronic supplementary material, figure S2. Two different horizontal planes have been stitched in (*e*1) and (*e*2). an, antennal nerve; ant, antenna; cn, cirral nerve; csc, cilia of the nuchal organ supporting cells; dp, dorsal peduncle of the MBs; nc, nuchal cavity; nn, nuchal nerve; np, main neuropil; nsn, neurosecretory neuropil; pn, palpal nerve; prc, photoreceptor cells; tc, tentacular cirrus; vp, ventral peduncle of the MBs. Further abbreviations: see the abbreviations list. Scale bars: 20 µm for all, 5 µm for inset in (*a*2).

We observed activity in 10 bilaterally symmetrical spots (figure 2), including four paired regions which we identified as the cell masses of the four presumed chemosensory organs (nuchal organs, palp, antennae and tentacular cirri). We also observed activity in another paired chemosensory region, the lateral region, as well as in the dorsal and ventral mushroom body (MB) regions. Finally, we saw activity in three bilaterally symmetric pairs of cells: one in the apical organ area, the eyefront cells and the fronto-dorsal cells. The lateral regions, the eyefront cells and the fronto-dorsal cells, so far undescribed, were named for the present study. Activity was also observed in the nuchal, palpal antennal and cirral nerves, which were thus included in the analyses.

To make sense of these signals, we first characterized the tissue context of the responding cells at the 6 dpf stage (figure 2; electronic supplementary material, figure S2). The *nuchal organs* (figure 2*a*; electronic supplementary material, figure S2A) lie posterior to the eyes and slightly more ventral. Nuchal cavities equipped with ciliated supporting cells can be recognized. The short nuchal nerves project posteriorly to the central neuropil and form a commissure. In the absence of clear anatomical boundaries, only proximity to the cavity and coactivation with the nerve can allow to attribute a cell to the nuchal cell mass. Most calcium activity was observed in a pair of cells, named ‘revolver cells’ due to their typical shape, but other cells next to the cavity were occasionally active. The *palps* (figure 2*b*; electronic supplementary material, figure S2B) lie ventrally, on each side of the mouth opening, and protrude only very little at this stage. Their mobile tip possesses clusters of cilia. The palpal nerves, short and thick, project to the central neuropil in proximity to the dorsal and ventral roots of the circum-oesophageal connectives. The palpal cell masses, delimited by membrane layers and developing coelomic cavities, constitute the largest cell masses in the head. Up to three pairs of active cells were observed rather in proximity to the nerves than to the tips. The *antennae* (figure 2*c*; electronic supplementary material, figure S2C) are slender, frontal organs. Cellular extensions and nervous fibres, but not cell bodies (electronic supplementary material, figure S3), are present inside the appendages, whose surface is equipped with clusters of cilia. The prominent antennal nerves, always identifiable in calcium recordings, project laterally to the central neuropil. The antennal cell masses, anatomically well delimited, are located at the base of the antennal appendages. Up to four pairs of active cells were observed throughout the cell masses. The mobile *tentacular cirri* (figure 2*d*; electronic supplementary material, figure S2D) possess clusters of cilia at their surface and nervous fibres. The cirral nerves project to the circum–oesophageal connectives, not to the central neuropil. The cirral cell masses, future cirral ganglia, are well delimited and occupy a lateral and posterior position in the head. Unlike the antennae, the cirral appendages contain cell bodies (electronic supplementary material, figure S3). Activity was recorded only in cells located at their base, amounting to a maximum of two pairs.

The *lateral regions* (figure 2*e*; electronic supplementary material, figure S2E) are well delimited, located between the palpal and cirral cell masses, close to the ciliary band called the akrotroch. Between one and three active cell bodies were observed in these regions, with no apparent neurite connection to the central neuropil. The MB regions consist of probably 15–20 cells each at this stage, organized around two neurite bundles called peduncles: a *dorsal* one, immediately lateral to the antennal nerve (figure 2*f*; electronic supplementary material, figure S2F), and a *ventral* one, ventral to the antennal nerve and dorsal to the palpal cell mass (figure 2*g*; electronic supplementary material, figure S2G). At 6 dpf, the strong condensation of MB cell nuclei characteristic of adult nereidid brains [53] is not yet apparent; hence, precise delimitation of MB cells is not possible. Nevertheless, active cells were observed in immediate proximity to the peduncles, and a coactivation with the peduncle was visible in ventral MB regions (inset in figure 2*g*1).

Single active cells were observed in the area of the *apical organ* (figure 2*h*; electronic supplementary material, figure S2H), an unpaired sensory organ present in annelid and other marine larvae and thought to be involved in their settlement, a crucial life cycle transition [54–56]. These two cells are close to the dorsal head surface, directly anterior to the akrotroch, in proximity to the neurosecretory neuropil known to be associated with the apical organ [57]. Their flask shape is typical for apical organ cells described in *Platynereis* at 2 dpf [58]. It should be noted that the cilia present dorsomedially at the head surface at 6 dpf do not belong to the apical organ but to another organ of unknown function, the dorsal ciliated pit (see electronic supplementary material, figure S2H1). A second pair of active cells was observed in a position immediately anterior to the eyes and therefore named *eyefront cells* (figure 2*i*; electronic supplementary material, figure S2I). Their activity, though observed only in a minority of animals, was prominent. A third pair, called *fronto-dorsal cells* (figure 2*j*; electronic supplementary material, figure S2J), was observed in a position slightly more dorsal and medial than the antennal nerves, and anterior to the central neuropil. These cells seem to have an axonal projection into the central neuropil, and their shape suggests an anterior cellular extension. A pair of tubulin-rich cells with a similar shape is present at the same position (figure 2*j*2), but we could not determine whether they are the same cells.

### 2.3. Nuchal organs, palps, antennae and tentacular cirri respond differentially to four chemical stimulants

**Figure 3.**
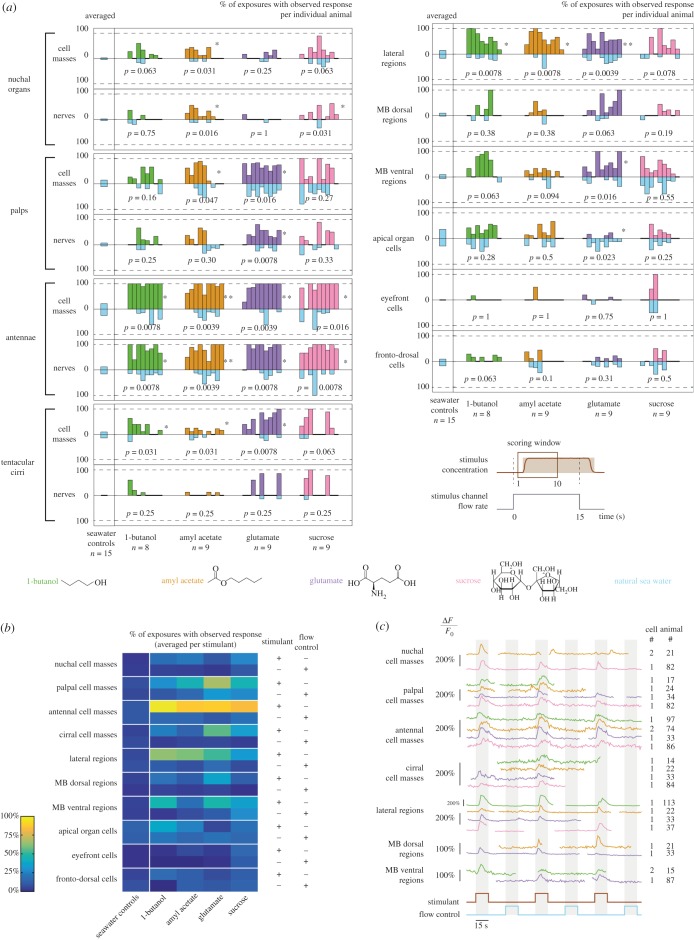
Calcium responses of the active regions to the four stimulants. (*a*) Fractions of exposures with observed response for individual animals, shown as mirror barplots for chemical stimulants (green, orange, purple, pink) and flow controls (light blue). For each region, an averaged value obtained in control experiments is shown on the left (separate light blue barplots). *p*-Values and significance levels of a Wilcoxon signed-rank test between responses to chemical stimulants and to flow controls are shown (*p < 0.05, **p < 0.005). (*b*) Fractions of exposures with observed response, averaged per stimulant. (*c*) Calcium activity traces of individual cells; calcium snapshots and the corresponding regions of interest are shown in the electronic supplementary material, figure S4. Trace colours correspond to the stimulants used.

Using four distinct chemical stimulants, we quantified the occurrence of responses for all regions and cells, in each animal, over a long time window following each stimulus onset (figure 3*a*,*b*; see Material and methods). The most obvious responses were those of the antennae, which responded systematically to each of the four stimulants. By contrast, the three other organs showed more differential responses. Nuchal organs were sensitive to amyl acetate and sucrose and, to a lesser degree, to 1-butanol, but did not seem to respond at all to glutamate. Palps responded to all compounds, but responses were observed for typically two-thirds of the exposures with glutamate, as opposed to about one-third for the other compounds, indicating that palps are particularly responsive to glutamate. In tentacular cirri, responses were seen frequently with glutamate and sucrose, and seldom with 1-butanol and amyl acetate, suggesting that glutamate and sucrose can elicit stronger responses. We performed an analysis of variance to quantify these differences (electronic supplementary material, table S1). Only cirral responses to glutamate versus amyl acetate differed significantly, but more differences would probably become apparent with an increased sample size (here, nine animals per compound). These results show that chemosensitivity in nuchal organs, palps and tentacular cirri is tuned to different types of stimulants.

Different causes seem to account for the non-systematic observation of responses in nuchal organs, palps and tentacular cirri. In nuchal organs, responses were only observed in about 30% of exposures. The fact that these responses were of high amplitude (typically Δ*F*/*F*_0_ = 100–150%) and occurred in large cells (diameter of 8–12 µm, as opposed to 5–9 µm in the other regions) excludes that they may have been omitted by our imaging, and suggests that they were conditional. In palps, responses were seen in about 60% of exposures on average. Since the responding cells were small (less than 6 µm in diameter) and because abundant muscle fibres and neurites produce calcium signals in this area, we attribute this percentage to technical difficulties in detecting the responses, rather than true biological variability. Responses in the tentacular cirri, whenever observed, occurred in a high fraction of exposures, but were nearly absent in some animals. Since they were overall the weakest responses (typically Δ*F*/*F*_0_ < 50%), we concluded that the cirri did respond to all stimulants, but the low amplitude of calcium signals did not always allow their detection.

In all organs, the responses were more frequent for the stimulants than for the flow controls (see statistical significance in figure 3*a*), which confirmed that the observed activity was chemically evoked. Control experiments without chemical stimulants showed that all organs had comparable levels of responses to natural seawater stimulations as here (figure 3*a*; see Material and methods), confirming that responses to flow controls in the present experiments did correspond to a non-chemically evoked activity. An overall increased activity of the palpal cell masses was nevertheless observed in the particular cases of glutamate and sucrose stimulation.

We next investigated chemically evoked responses at the single-cell level rather than for the entire organs. In palps, antennae and tentacular cirri, we found that most cells which responded to a stimulant did so systematically and responded only after its onset. Examples are shown for each stimulant in figure 3*c* (calcium signal snapshots and regions of interest (ROIs) are shown in electronic supplementary material, figure S4; more examples of activity traces can be found in Chartier [34, ch. IV]). These findings indicate that these organs are indeed able to directly detect the onset of such stimulants. In nuchal organs, sucrose was the only stimulant for which we could observe reproducible responses, though only in a single animal. Two examples of activity traces are shown in figure 3*c*: the first is representative of responses overall observed in nuchal organs (only some of the calcium transients correlate with a stimulus onset), and the second shows repeatable responses observed in this single animal. Hence, while the nuchal organs are able to directly respond to the onset of at least some stimulants, such responses do not seem to be robust in our assay.

### 2.4. Chemosensory responses of the mushroom body regions and a newly identified lateral region

Similar to nuchal organs, palps, antennae and tentacular cirri, the lateral and MB regions showed an enhanced activity directly related to the onset of chemical stimuli (figure 3*a*,*b*), which identifies these regions of the early differentiated *Platynereis* brain as part of the chemosensory circuits. Responses were observed in about 65% of exposures for the lateral regions, 40% for the ventral MB regions and 15% for the dorsal MB regions. The lateral regions responded to all compounds, though slightly less to sucrose, indicating a broad chemosensitivity as for the antennae. The MB regions showed more differential responses, with the ventral ones being more responsive to 1-butanol and glutamate than to amyl acetate and sucrose, and the dorsal ones being mostly responsive to glutamate. Flow controls confirmed the chemically evoked nature of responses in the three regions. Only responses of the ventral MB regions upon sucrose stimulation were an exception; in this particular case, another factor than the stimulant may have led to an overall increased activity (three times higher than in control experiments). We found examples of chemically evoked single-cell responses to all stimulants for the lateral regions, to amyl acetate and glutamate for the dorsal MB regions, and to 1-butanol and glutamate but not sucrose for the ventral MB regions (figure 3*c*; calcium signal snapshots and ROIs are shown in electronic supplementary material, figure S4). This confirmed that the lateral regions respond to all stimulants, and the two MB regions to at least some of them.

By contrast, no evidence for stimulant-specific responses could be found in the apical organ cells, the eyefront cells or the fronto-dorsal cells.

### 2.5. Responses are observed with a delay in the lateral regions and mushroom body regions compared with the major chemosensory organs

**Figure 4.**
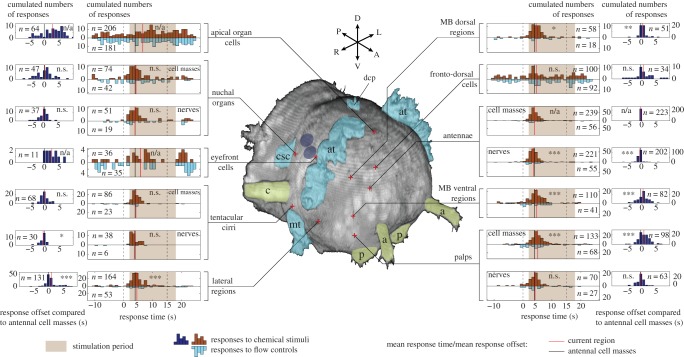
Distribution of response times with respect to the stimulation period. Relative response times, with the pump trigger time of the stimulus taken as *t* = 0 s, are shown in the inner graphs. The total numbers of available responses are indicated. Student's *t*-test was used to compare the mean of the distributions with the mean response time of the antennal cell masses. Delays over the antennal cell masses are shown in the outer graphs. The total numbers of responses for which a delay could be calculated (responses which co-occurred with an antennal response) are indicated. Student's *t*-test was used to compare the mean delay with 0 (n.s. = non-significant, *p < 0.05, **p < 0.005, ***p < 0.0005, n/a, not applied). The three-dimensional head reconstruction is obtained from the same anatomical stainings as shown in figure 2. Eyes (dark blue ovals), ciliated structures (cyan) and head appendages (green) are highlighted: a, antenna; at, akrotroch; c, tentacular cirrus; csc, cilia of the nuchal organ supporting cells; dcp, dorsal ciliated pit; mt, metatroch; p, palp.

Following the observation that seven regions were activated by stimulants, we set out to determine when exactly their responses occurred with respect to the window of exposure. The cumulated distributions of all response times were calculated for each region, by pooling experiments involving all four stimulants (figure 4, inner graphs). The response times correspond to the beginning of calcium transients (see Material and methods).

For the nuchal organs, palps, antennae and cirri, the vast majority of responses took place within 5 s following the stimulus onset. The observed variability in these response times was at least partly attributable to variable stimulus onsets compared with the pump triggering times (electronic supplementary material, figure S1C). Activity outside of the stimulation period was seen more often in the nuchal organs than in the three other chemosensory organs, in agreement with the more erratic responses observed in single-cell activity traces (figure 3*c*).

For the lateral regions and MB regions, responses were likewise observed predominantly following stimulus onset, but the mean response times were slightly delayed. Taking as a reference the antennal cell masses' responses—the most abundant and robust ones—we saw indeed a statistically significant delay in the order of 1 s for the mean response time of the lateral regions and of 0.75 s for the two MB regions, but no significant delay or lead for the nuchal organs and cirri (figure 4, inner graphs). The palps did respond as fast as the antennae, because the delay observed for the cell masses was absent for the nerves.

Complementarily, the offsets of response times with respect to the antennal cell masses were assessed in individual animals, by calculating the actual offset for each response of each region (see Material and methods). The cumulated distributions of these offsets confirmed the statistical significance of the observed delays (figure 4, outer graphs). A rather wide distribution of offsets was visible for the palpal cell masses and the ventral MB regions, as opposed to the other regions responsive to stimulants. For the lateral regions, the offset distribution seemed to be bimodal, with a main peak at around +1 s and a minor one at around +5 s (15% of the responses). A small but significant lead was observed for the antennal nerves over the antennal cell masses (in the order of 0.3 s). The observation that the lateral regions, the two MB regions as well as the palpal cell masses responded with some delay with respect to the overall synchronized responses of the chemosensory organs was robust when offset distributions were alternatively calculated against the cirral cell masses, cirral nerves or palpal nerves (data not shown).

Activity in the apical organ cells and eyefront cells was rather uniformly distributed, irrespective of the chemical stimulation period. For the fronto-dorsal cells, the coexistence of a uniform activity and a peak of responses synchronized to the antennal ones suggested that these cells may show both specific and unspecific responses.

### 2.6. The apical organ cells show a periodic activity, synchronized with the eyefront cells

**Figure 5.**
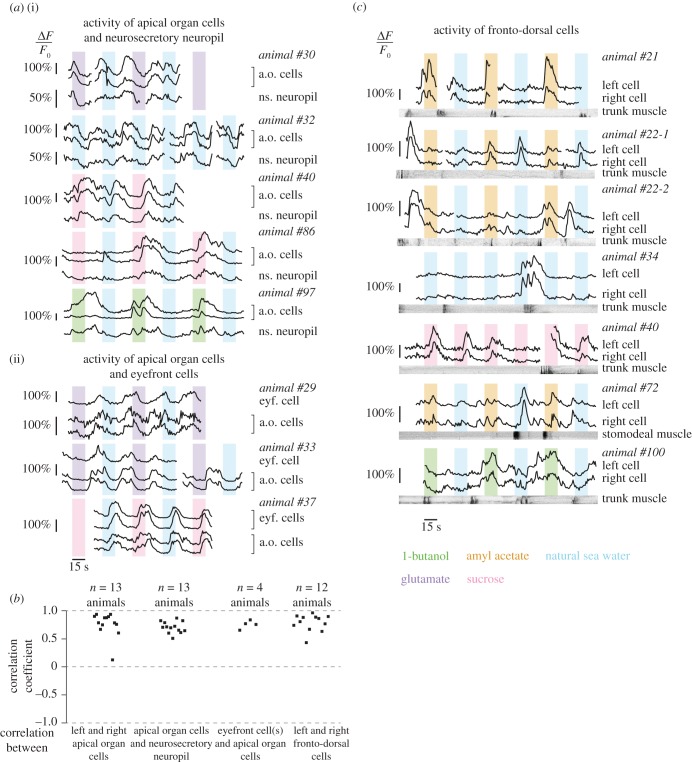
Calcium activity of the apical organ cells, eyefront cells and fronto-dorsal cells. (*a*) Individual calcium traces showing synchronization between apical organ cells and the neurosecretory neuropil (i) and between eyefront cells and apical organ cells (ii). The stimulations are colour-coded. (*b*) Correlation of the calcium signals between cells. (*c*) Individual calcium traces showing responses of the fronto-dorsal cells in relation with stimulations (colour-coded) and with locomotor activity (kymographs). Calcium snapshots and the corresponding regions of interest for (*a*) and (*c*) are shown in the electronic supplementary material, figure S5. Dark parts of the kymographs correspond to movement.

We further analysed single-cell activity in apical organs cells, eyefront cells and fronto-dorsal cells, to better understand why this activity was prominent in our experiments, though not obviously chemically induced (figure 5). Activity traces reveal that in 13 out of 35 animals, apical organ cells had slow, large-amplitude calcium fluctuations, as shown in figure 5*a* (for calcium signal snapshots and ROIs, see electronic supplementary material, figure S5). These fluctuations were synchronous between both cells, as well as with the closely located neurosecretory neuropil (figure 5*b*). We found that the eyefront cells, though rarely active, were always synchronized with the apical organ cells (figure 5*a*,*b*), which suggests that these two pairs of cells are interconnected. The calcium fluctuations’ period, typically 35 s (animals #29, 30, 33, 37, 40), matches the periodicity of the alternated stimulations with and without chemical stimulant (see Material and methods). Although the fluctuations had variable phases compared with the stimulations, they could thus have been entrained by the flow stimulations, independently of chemical stimulants; in fact, they can take place in the absence of chemical stimulant (animal #32). Yet, additional experiments, either without flow patterns or with a different periodicity of flow patterns, would be needed to exclude that these cells had an intrinsic periodic rhythm which accidentally matched the flow period used here. Nevertheless, in two animals, a different pattern of activity was observed, with beginnings of calcium transients correlating with the onsets of chemical stimuli and resulting in similar fluctuations with a period of approximately 70 s, i.e. double (animals #86, 97). This suggests that apical organ cells could still be responsive to chemical stimulants, at least to 1-butanol and sucrose. On the whole, the present experiments do not suffice to conclude whether or not chemical stimulants trigger the oscillating calcium activity in the apical organ cells and eyefront cells.

### 2.7. Activation of the fronto-dorsal cells partially coincides with stimulus onset or termination of locomotor activity

In all experiments, activity was highly synchronous between the two fronto-dorsal cells (figure 5*b*). A closer look at single-cell activity confirms that these cells do show occasional responses to the onsets of chemical stimuli (figure 5*c*, animals #21, 22-1, 40; for calcium signal snapshots and ROIs, see electronic supplementary material, figure S5), as previously suggested by the distribution of response times. Responses to the onset of the flow controls were also observed (animal #22-1). Besides, activation of these two cells was seen to follow muscle contraction in the trunk or stomodeum in several animals (animals #21, 22, 40, 72, 100). The existence of overlapping calcium transients when an episode of movement and a stimulus onset rapidly follow each other (animals #21, 22-2, 34) suggests that both types of activity may add up.

## 3. Discussion

### 3.1. *Platynereis* possesses four types of head chemosensory organs

Our calcium imaging experiments reveal chemically evoked responses to four types of chemical compounds in nuchal organs, palps, antennae and tentacular cirri (figure 3*a,b*). Single-cell responses (figure 3*c*) immediately follow the onset, not the offset, of the chemical stimuli, and are synchronous for the four organs (figure 4). The lateral regions may constitute a fifth type of primary chemosensory organ, as suggested by their proximity to the surface (figure 2*e*) and their high activity upon chemical stimulations (figure 3*a*). However, the absence of externally obvious sensory structures associated with them (figure 2*e*) as well as their systematic response delay compared to the four other organs (figure 4) would speak against that, hence this question deserves further investigation.

In palps, antennae and tentacular cirri, responsive cell bodies were only seen at the base of the appendage, hence at least part of them must correspond to sensory neurons that extend a long dendritic process through the appendage until the actual site of chemical detection; in fact, such processes were sometimes visible in the calcium signal (e.g. electronic supplementary material, video S1). However, part of the active cells may well have been interneurons. In nuchal organs, only two cell types are present around the sensory cavity: sensory neurons which extend a dendritic process into the cavity and ciliated supporting cells [59,60], while interneurons described in *Platynereis* at 3 dpf are distant from the cavity [61]. The revolver cells are thus clearly sensory neurons (see morphology in figure 2*a*), and so are probably the rare other responsive cells observed close to the cavity.

We showed that nuchal organs, palps and tentacular cirri, unlike the antennae, respond differentially to the compounds, which suggests a specialization of these organs in terms of chemosensory repertoire. Our few attempts to test for functional differences between cells belonging to the same organ, using two stimulants per animal, were so far inconclusive (data not shown). Nevertheless, it is likely that at least antennae and palps could be capable of chemosensory discrimination, due to the high number of cells they possess.

Antennae appear to be central in *Platynereis*' chemosensation and are probably responsible for the general identification of chemical cues. It came as a surprise that their responses were by far more systematic than those of the nuchal organs, because the latter are generally thought to be important for annelid chemosensation, but not the former [24,36]. Palps, which are located close to the mouth and were more strongly activated by the amino acid and the sugar (figure 3*a*,*b*), may be specialized in the detection of directly food-related chemical cues, as is hypothesized in spionid annelids [22,38,62]. These highly musculated appendages, which adult *Platynereis* use for prehension of food items (TF Chartier 2015, personal observations), could also serve in the contact chemoreception of hydrophobic compounds, whose importance is often underestimated in marine animals [63,64]. The tentacular cirri sometimes showed separate response times between the left and right side (data not shown). It is likely that these long organs, which extend in different directions in adults, can provide relevant spatial information about the localization of chemical cues. These tactile organs are also photosensitive and involved in the shadow reflex in *Platynereis* [65]. Hence, their role is probably to collect general multisensory information about objects approaching the head, in order to produce immediate, coarse responses. Finally, there is little doubt that the highly conserved nuchal organs play an important sensory role in annelids; hence, it is likely that we did not test the most relevant cues for them.

*Platynereis* detects 1-butanol and glutamate, which corroborates behavioural observations made long ago in *Nereis* [44]. Amino acids, such as glutamate, are general chemical cues in aquatic environments, as is known in fish and crustaceans [66,67]; other known cues such as nucleotides, steroids and bile acids [68] should be tested in future experiments. The stimulants were presented here at 10 µM, but the presence notably in antennae of responses that slightly precede the earliest possible onset of this concentration (figure 4; electronic supplementary material, figure S1C) suggests that lower concentrations can be detected. The fact that 1-butanol and amyl acetate, which are odorant molecules for humans, act as distance cues in the water for *Platynereis* provides support to the view of Mollo *et al*. [1, section 8] that the traditional categories of ‘olfactory molecules’ and ‘taste molecules’ should be abandoned.

Our results in *Platynereis* suggest that marine annelids possess head chemosensory organs with distinct roles, adapted to sets of chemical cues relevant in different situations (feeding, escaping, reproduction, etc.), similar to what is known from crustacean chemoreception [69]. Antennae, which seem to be the main chemosensory organs in *Platynereis*, are present in a vast majority of annelid taxa [70] and may thus be of general importance in annelid chemosensation. Though we did not test it here, a distributed chemosensitivity of the body surface is likely for annelids, as suggested by previous behavioural and anatomical studies [19,26,71,72].

### 3.2. Annelid mushroom bodies as possible chemosensory integration centres

Mushroom bodies (MBs), which have long been described in annelid brains [25,27], have a high anatomical similarity with their homonyms in insects [73]. In fact, similar structures are found in several protostome phyla including flatworms, nemerteans and onychophorans [74], which suggest that they may have been inherited from the last common protostome ancestor's brain. In insects, MBs are the place where associative memories are formed, notably with odour stimuli [75–77]. In mammals, this role is endorsed by the pallium [78–80], which includes cortex and hippocampus, and develops from neuroectodermal brain regions expressing similar combinations of transcription factors as in *Platynereis* [81]. We observed cells located in the dorsal and ventral MB regions responding specifically to several chemical stimulants (figure 3*a,c*). In particular for the ventral regions, coactivation with the MB peduncles proved that these responsive cells indeed belonged to the MBs (figure 2*g*1, inset). Our observations represent the first physiological data available for annelid MBs and firmly establish them as part of the chemosensory system. MB cellular responses were delayed compared to the four major organs (figure 4) and may have stemmed from sensory interneurons. This would be in line with a presumed role of MBs in the representation and integration of chemical cues also in the annelid brain.

### 3.3. Apical organ cells, eyefront cells and fronto-dorsal cells may be part of the chemosensory circuits

We have identified three distinct pairs of cells in the head whose activity may be partially linked to chemical stimulations. Activity in the apical organ cells did correlate with the onset of chemical stimulations in at least two animals (figure 5*a*). This preliminary evidence for chemosensitivity calls for further exploration, as apical organs are likely important in the settlement and metamorphosis of marine larvae in general, which they are thought to trigger via the detection of environmental chemical cues [54–56]. The long duration of calcium transients in these cells, as well as their proximity to and coactivation with the neurosecretory neuropil (figure 2*h*, figure 5*a,b*), suggest a neurosecretory nature. One can hypothesize that upon detection of appropriate settlement cues, these two cells would adopt periodic patterns of neurosecretory activity as observed here, signalling to the animal that settlement can start. Alternatively, calcium fluctuations in these cells may have been merely entrained by the changing flow patterns, as would also be consistent with our data. However, we do not favour this latter hypothesis, because their prominent activity should then have been observed in most animals, not in one-third of them. Whenever the eyefront cells were visible, their activity was tightly synchronized to that of the apical organ cells; hence, both pairs of cells may belong to a common circuit involved in neurosecretion and/or larval settlement. The pair of fronto-dorsal cells, which are probably neurons due to their morphology and activity patterns (figure 2*f*; electronic supplementary material, figure S5), were seen to respond after at least three types of events: chemical stimulations only, chemical stimulations and flow stimulations, locomotor episodes (figure 5*c*). Hence, their responses to chemical stimulants were not primary sensory responses. These neurons may have an inhibitory effect, their role being either to prevent a locomotor reaction to external stimuli such as chemical cues, or to stop an ongoing locomotor episode. We hypothesize that they form part of a general circuit for locomotor inhibition receiving inputs from different sensory modalities. As such, they may represent a non-specific part of the chemosensory circuits.

### 3.4. Variability of responses: biological and technical factors

While antennal responses were strong and systematic for all animals and all compounds, responses in palps and tentacular cirri could not be observed in all animals or for all exposures (figure 3*a*). Since single-cell responses, though of lower amplitude than in antennae, were always robust (figure 3*c*), we interpret this fact as a consequence of their response amplitude sometimes falling below our detection threshold. We conclude that these two organs do detect these stimulants, which a more targeted imaging would allow to verify. By contrast, single-cell calcium responses to stimulants in the nuchal organs were hardly repeatable (figure 3*c*). The typically high amplitude of these responses, whenever observed, rules out an issue of detection threshold. While we cannot exclude that other compounds may elicit systematic responses, it seems that nuchal organs respond to chemical stimulants in a more conditional manner, though we were so far unable to tell what influences their responsiveness. For the four chemosensory organs, the nerve was seen to respond with a slight lead over the cell mass (figure 4), even though the former is located anatomically downstream of the latter. We interpret this as an effect of geometry, with local calcium concentrations increasing faster in the axons than in the somata. Finally, in most active regions, a majority of responses were visible only on one side of the head, for example, in figure 2*d*1 or *g*1 (see quantification in electronic supplementary material, figure S6). Because the imaging planes can be slightly tilted, we regard this as an artefact of imaging, occurring when a weakly responsive nerve or cell body on one side is located between two consecutive optical planes and fails to be detected. However, we are confident that all responses are indeed bilateral, notably because no anatomical asymmetry is known for the head and because the regions where most non-bilateral responses are seen are the most difficult to image and show the weakest responses (palpal and cirral nerves, MB dorsal and ventral regions; see electronic supplementary material, figure S6). Besides, an activity can appear non-bilateral for the cell masses, while it is clearly bilateral in the corresponding nerves, as is obvious for the nuchal organs and antennae (electronic supplementary material, figure S6).

### 3.5. Sensory cell anatomy and physiology is different between nuchal organs and head appendages

Based on our functional imaging data, nuchal organs seem to possess a different physiology than the three other chemosensory organs, and incidentally, they are known from electron microscopy studies to possess different types of sensory cells than palps, antennae and tentacular cirri. While cells of the three appendages have sensory cilia that traverse the cuticle and come directly in contact with the environment [23,26,31,39,82–84], sensory cilia of nuchal organ cells sit in a fluid-filled sensory cavity shielded from the environment by specialized cuticular or microvillar layers [59,60,85,86]. If these layers affect the diffusion of molecules, changes of chemical composition in the fluid environment may be effective with some delay inside the cavity compared with the surface of appendages. The nuchal organs' shielded anatomy suggests that they may detect the global presence and concentration of ambient chemical cues, but not capture their rapid concentration dynamics, as the three appendages probably do. A role of these organs in inter-individual communication and the detection of pheromones constitute a good working hypothesis.

### 3.6. A microfluidics set-up for immobilization and targeted stimulus delivery

The use of microfluidics for *in vivo* experiments offers several advantages over the immobilization methods used so far with *Platynereis* juveniles and larvae: gluing, MgCl_2_ or mecamylamine paralyzing, low-melting agarose embedding or slide-coverslip mounting. The present device allows both a reliable animal immobilization without any chemical agent potentially interfering with the animal's physiology and an ecologically relevant exposure to chemical stimulants. Moreover, stimulant exposures are precise and repeatable, which is key for probing single-cell activity.

Further experiments can be performed with the same set-up, notably testing discriminatory abilities of the chemosensory organs between either two concentrations or two stimulants. Any other waterborne stimulus such as pH, salinity, O_2_ or CO_2_ levels, or even small solid particles, can be used and possibly combined with a precise pharmacological treatment. Adding a dye to the stimulus solution would allow a more precise monitoring of stimulus timing, though all dyes we have tested were improper in that they triggered antennal chemosensory responses. Whole-head recordings at higher spatial and temporal resolutions could be obtained using a light-sheet microscope (as in [87]). The microfluidic device is suited for experiments on 4–7 dpf animals and can easily be adapted for younger stages. Beyond 7 dpf, muscles, hence movement artefacts, are too strong, and either a more elaborate trap or a drug treatment would be needed to achieve immobilization. The trap design could also be adapted for other marine larvae or small organisms, some of which are suited for microfluidic experiments (TF Chartier 2015, personal observations). Though our conclusions would not have been altered, absorption issues documented for PDMS devices may have reduced effective stimulant concentrations [88]; hence, alternative materials such as COC [89] or sTPE [90] may be preferred to PDMS for future experiments. Microfluidic set-ups have been successfully used to explore neuronal and motor activity in nematodes and fish larvae [4,91], and our study shows that similar experiments are possible with *Platynereis* juveniles.

### 3.7. Early *Platynereis* juveniles as a model for the study of annelid chemosensory systems

*Platynereis* sensory organs and nervous systems are representative for annelids, and early juveniles already possess all types of adult chemosensory organs. Our results show that whole-head activity upon precisely controlled chemical stimulations can be imaged in early juveniles. Hence, these juveniles can be used to test the general physiology of annelid chemosensory organs, as monitored by any fluorescent reporter. The availability of a cellular resolution expression atlas [41] in combination with single-cell sequencing and mapping onto the atlas [92,93] will enable efficient identification of candidate chemoreceptor genes. Candidates would then be validated by combining knockouts [94] of receptor proteins and functional imaging, and possible *in vitro* deorphanization of receptors [95]. We have demonstrated that single cells, such as the eyefront cells, fronto-dorsal cells or apical organ cells, can be identified based on their calcium activity patterns (figures 2 and 3). Mapping functional imaging data as acquired here onto the gene expression atlas will allow thorough characterization of such cells. Furthermore, the feasibility of connectomics in *Platynereis* has been proved at the 3 dpf stage [43,44,57,61], and a connectomic effort at 6 dpf is ongoing, which could allow to add circuit information to the molecular and functional data presented here. It will be interesting in the future to investigate *Platynereis*' abilities for sensory integration and associative learning, whose neuronal correlates could be directly studied with the present set-up. Finding an involvement of annelid MBs in associative learning would be of particular interest for the comparative neurobiology of learning.

## 4. Conclusion

We have established that nuchal organs, palps, antennae and tentacular cirri are chemosensory organs in *Platynereis*, responding to an alcohol, an ester, an amino acid and a sugar. This conclusion is likely to extend to annelids, for which similar sensory cells have been detected in electron microscopy. Our results show a capability to differentially respond to multiple chemosensory cues, which opens the possibility of complex chemosensory integration. With our findings, we establish 6-day-old *Platynereis* juveniles as an experimental system for the chemosensory physiology of marine annelids.

## 5. Material and methods

### 5.1. Device fabrication

Standard soft lithography was used to fabricate the mould [96]. The photomask was designed with AutoCAD (2014 free student version, Autodesk, Inc.) and printed at a resolution of 25 400 dpi by an external company (Selba S.A., Versoix, Switzerland); the source file is available as .dwg file in the electronic supplementary material. The trapping channel's width linearly decreases from 150 to 75 µm at its end, which constitutes the trap. The mould with a uniform height of 60 µm was obtained by spin-coating a silicon wafer (4 inches; Siltronix, France) with a negative photoresist (SU-8 2050, MicroChem Corp., Newton, MA, USA). Devices were produced by pouring onto a mould and curing at 65°C for a minimum of 4 h a prepolymer mixture of polydimethylsiloxane (PDMS, Sylgard 184 silicone elastomer kit, Dow Corning Corp.) with a 1 : 9 ratio of curing agent. PDMS blocks were then irreversibly bound to a 0.17 mm glass coverslip (#1871, 24 × 50 mm, Carl Roth GmBH, Germany) by a 1 min treatment in a plasma oven (Femto, Diener electronic GmbH & Co. KG, Germany). For a detailed fabrication protocol, see chapter IV and appendix E in Chartier [34].

### 5.2. Experimental set-up and procedure

Filtered natural seawater obtained with 0.22 µm sterile filters (Millipore), plastic syringes (Luer Plastipak, BD, USA), metallic needles (Microlance #20, 302200, BD, USA) and polytetrafluoroethylene tubing (PTFE, TW24, inner diameter 0.59 mm, Adtech Polymer Engineering Ltd, UK) were used in all experiments. A 1 mM stock solution was prepared weekly for each chemical stimulant. Working solutions at 10 µM were prepared on the day of the experiment, and syringes loaded with these solutions were placed in the experimental room 1 h before starting. All solutions were kept at 18°C and handled in glassware, because preliminary experiments had revealed that the animals may detect dissolved substances from plastic containers such as Falcon tubes. Water streams were generated in a laminar flow regime (7 mm s^−1^, Reynolds number ≈0.5). Flow rates of 8.33, 33.33 and 41.66 µl min^−1^ were used in the channels (figure 1*d*), with the total flow rate kept constant (50 µl min^−1^) to minimize pressure changes experienced by the animal. The device was operated by push-pull pumps (AL4000-220Z, WPI Germany GmbH), which were computer-automated via Micro Manager (v. 1.4.21, [97]). Image acquisition and stimulus delivery were synchronized with Auto Mouse Click (MurGee.com), a software for automated mouse actions. A customized metallic chip holder was built to hold the fragile device and facilitate its observation under an upright microscope. For details on the pump automation and the pumping programmes, see chapter IV and appendix D in Chartier [34].

### 5.3. Imaging

GCaMP6s fluorescence excited at 488 nm was detected by a hybrid detector (HyD) set to photon-counting mode in a Leica TCS-SP8 confocal microscope, equipped with a 40× (NA 1.1) water-immersion objective (water was preferred to oil for experimental convenience). Transmitted light images were recorded with a classical PMT detector, from the same excitation light as GCaMP. The head region was imaged in 12 horizontal optical sections (pinhole opened at 6.4 Airy units) sampling the whole volume at 5 µm intervals. To balance potential biases due to increased signal loss with tissue depth, approximately half of the animals were imaged from the dorsal side and the other half from the ventral side, thanks to adequate trapping. Images were acquired by confocal scanning at 8 kHz (resonant mode, phase X correction 1.32, laser powers 6–28 µW, pixel dwelling time 50 ns).

### 5.4. Calibration experiments

A green dye (tartrazine, E102) was dissolved in the two side streams to visualize their moving boundaries. Transmitted light intensity from a 633 nm laser illumination was measured at 10 Hz resolution in a square region of interest, constant in size and position, located just upstream of the animal's head (brown rectangle in figure 1*g*). Minimal and maximal intensities, which according to the Beer–Lambert law corresponded, respectively, to the absence of stimulant and maximum stimulant concentration, were normalized between 0 and 1. Edge detection allowed to quantify the beginning and ending of stimulation onsets and offsets (electronic supplementary material, figure S1C). Measurements were made successively with eight trapped animals (four experiments per animal).

### 5.5. Animal preparation and handling

*Platynereis* juveniles were obtained from a permanent culture following Hauenschild & Fischer's breeding protocol [98] and kept at 18°C with 16 L : 8 D cycles. Calcium imaging experiments were conducted at 18–20°C, between 142 and 177 h post-fertilisation (hpf), at various times of the day and night. For each chemical stimulant, animals coming from at least two distinct batches were imaged. The calcium reporter GCaMP6s [99] was transiently and ubiquitously expressed by microinjecting *Platynereis* eggs with mRNA (1.000 ng µl^−1^) between 1 hpf and the first cleavage. Capped and polyA-tailed mRNAs were synthesized with the mMESSAGE mMACHINE T7 Ultra Kit (Life Technologies) from a vector obtained from the Jékely lab (pUC57-T7-RPP2-GCaMP6 described in [43]). After micro-injection, eggs were kept in filtered natural seawater and culture conditions remained unchanged. The device was washed with filtered natural seawater for 4 min before every new animal was manually introduced with a syringe. Each animal was allowed to rest for 5 min before the experiments, and successive experiments on one animal were performed at 5–10 min intervals. After the experiments, the animal could be recovered without damage for potential further observations, by gently flushing it out of the device.

### 5.6. Response assessment

Cellular or nerve responses were assessed with the human eye from raw recordings. In the absence of specific genetic markers, the attribution of a responsive cell to a region relied on its position, guided by anatomical landmark recognition based on precise reference immunostainings (figure 2; electronic supplementary material, figure S2). A given region was considered to respond whenever at least one cell was seen to respond in at least one of the region's two bilaterally symmetric parts—for a given nerve, whenever at least one of the two bilaterally symmetric nerves was seen to respond. The bilaterality of observed responses is quantified in the electronic supplementary material, figure S6, where every response has been classified as ‘bilateral’, ‘left only’ or ‘right only’, and the relative fractions of such responses were plotted as barplots for each region. The time of occurrence of a response was defined as the beginning of the corresponding calcium transient, and the threshold of visual detection of such an event corresponded to a signal-to-noise ratio of approximately 3 : 1. To be noted: a permanent activity of the eye photoreceptor cells was induced by the 488 nm laser illumination. All response scorings were performed twice at a 6-month interval by the same person; the resulting data are available as .xlsx file in electronic supplementary material, table S2.

### 5.7. Quantification of activity

#### 5.7.1. Occurrence of responses (figure 3)

A scoring window was defined, starting with the earliest possible onset of the stimulant (1 s after the pump trigger, see electronic supplementary material, figure S1C) and lasting 9 s. Responses of the regions were scored for each stimulus exposure, with a 1 counted if at least one response was seen during the scoring window and a 0 otherwise. The fractions of exposures with an observed response were calculated for each animal and each region, separately for exposures to stimulants and flow controls (figure 3*a*). The averages of these fractions for each stimulant are shown as a heatmap (figure 3*b*). For each set of animals assayed with a given stimulus, e.g. for the nine animals tested with glutamate, a Wilcoxon signed-rank test (significance level *α* = 0.05) was performed for each region to determine whether the fraction of responses over exposures statistically differed between the chemical stimulant and its flow controls (figure 3*a*). To quantify response occurrences in the absence of chemical stimulation, control experiments were run: 15 of the animals assayed with chemical stimulants had been beforehand imaged in one experiment with both side channels containing filtered natural seawater only, prior to any introduction of chemical stimulant in the device. The response occurrences were quantified as described above, and a global fraction of observed responses over the number of exposures was calculated for each region by pooling responses of the 15 animals (figure 3*a*,*b*).

#### 5.7.2. Distributions of relative response times and response offsets (figure 4)

All responses of all 35 animals were pooled for each region, and the cumulated distributions of their times of occurrence with respect to the stimulation period were calculated. Student's *t*-test (*α* = 0.05) was performed to determine whether the mean of each distribution differed from the mean response time of the antennal cell masses (all means were calculated over the 9 s scoring window defined for figure 3). Additionally, for each individual response that co-occurred with a response of the antennal cell masses within this window, the time offset between the two was calculated. The cumulated distributions of these offsets for each region are shown as barplots in figure 4 (outer graphs) for responses to the chemical stimuli only, not to the flow controls. Student's *t*-test (*α* = 0.05) was performed to determine whether the means of these offsets differed from 0, i.e. whether these regions responded at a different time than the antennal cell masses.

#### 5.7.3. Single-cell activity traces and kymographs (figure 3*c*, figure 5*a,c*)

Movement artefacts on the raw calcium recordings were first corrected in ImageJ (v. 1.50a) using the plugin StackReg [100] with rigid body transformations. Whenever needed, several parts of the recordings were registered individually and the traces obtained from each of them subsequently reassembled. Mean fluorescence intensity was then calculated from regions of interest (ROI) drawn manually in ImageJ. Further data analysis was done in MATLAB (2014a student version, The MathWorks, Inc.). Traces were plotted as Δ*F*/*F*_0_, with *F*_0_ calculated as the mean fluorescence value over a 10 s time window during a resting state, i.e. outside of stimulation periods. Muscle contractions (figure 5*c*), which are likewise reflected by calcium activity, were quantified in the same way from ROIs drawn manually on part of the trunk or stomodeal muscles, and plotted as kymographs instead of curves, with darker parts corresponding to higher fluorescence levels.

### 5.8. Immunohistochemistry

Animals collected at the 6-day stage (precisely 144 hpf) were fixed in 4% PFA and 0.1% Triton X-100. Tubulin structures were marked with a monoclonal mouse antibody against alpha-acetylated tubulin (Cat# T6793, Sigma-Aldrich GmbH, Germany, 1 : 250 dilution) and an Alexa 488 secondary antibody (Jackson Laboratories, USA, 1 : 500 dilution). Nuclear DNA was stained with DAPI (1 : 1000 dilution) and membranes with mCLING–ATTO 647N (Synaptic Systems GmbH, Germany, 1 : 50 dilution). DAPI, tubulin and mCling fluorescence was excited at 405, 488 and 633 nm, respectively, and recorded with a Leica TCS-SP8 confocal microscope equipped with a 63× glycerol-immersion objective. For details, see chapter III in Chartier [34].

## Abbreviations list

ac: anal cirrus; adc: anterior dorsal tentacular cirrus; an: antennal nerve; ant, a: antenna; at: akrotroch; avc: anterior ventral tentacular cirrus; bv: blood vessel; cc: circum-oesophageal connectives; ch: chaetae; cn: cirral nerve; cp: ciliary photoreceptor; csc: cilia of the nuchal organ supporting cells; dcp: dorsal ciliated pit; dp: dorsal peduncle of the mushroom bodies; drcc: dorsal root of the circum-oesophageal connectives; ec: pigmented eye cup; g: gut; gc: glandular cell; j: jaw; ld: lipid droplet; mo: mechanosensory organ; mt: metatroch; nc: nuchal cavity; nco: nuchal commissure; nn: nuchal nerve; np: main neuropil; nsn: neurosecretory neuropil; pa, p: palp; pcc: palpal coelomic cavity; pdc: posterior dorsal tentacular cirrus; pm: palpal muscle; pp: parapodia; prc: photoreceptor cells; pvc: posterior ventral tentacular cirrus; rpn: roots of the palpal nerve; sog: sub-oesophageal ganglion; st: stomodeum; stn: stomodeal nerve; tc, c: tentacular cirrus; vp: ventral peduncle of the mushroom bodies; vrcc: ventral root of the circum-oesophageal connectives.

## Supplementary Material

Chartier et al 2018 - Supplementary figures and tables

## Supplementary Material

Chartier et al. 2018 - Supplementary table 2 - Scoring of responses_machine-readable

## Supplementary Material

Chartier et al 2018 - Supplementary file - Design of microfluidic design

## Supplementary Material

Chartier et al. 2018 - Supplementary video 1 - Activity in the antennae and MB ventral regions - speed x5

## Supplementary Material

Chartier et al. 2018 - Supplementary video 2 - Activity in the lateral region - speed x5

## Supplementary Material

Chartier et al. 2018 - Supplementary video 3 - Oscillatory activity in the apical organ cells - speed x15.avi

## Data accessibility

The datasets supporting the conclusions of this article, as well as videos showing examples of calcium activity, have been provided as the electronic supplementary material. Raw calcium recordings are available on demand.
