# Supplementary material for: Whole-head recording of chemosensory activity in the marine annelid Platynereis dumerilii
Source: Open Biol. 2018 Oct 31;8(10):180139. doi: 10.1098/rsob.180139 (PMC6223215; doi:10.1098/rsob.180139)
Supplement: Chartier et al 2018 - Supplementary figures and tables [file rsob180139supp1.pdf]

Supplementary material for :

Chartier TF, Deschamps J, Dürichen W, Jékely G, Arendt D 2018, Whole-head recording of chemosensory activity in the marine annelid *Platynereis dumerilii*, *Open Biology*
(doi: 10.1098/rsob.20180139)

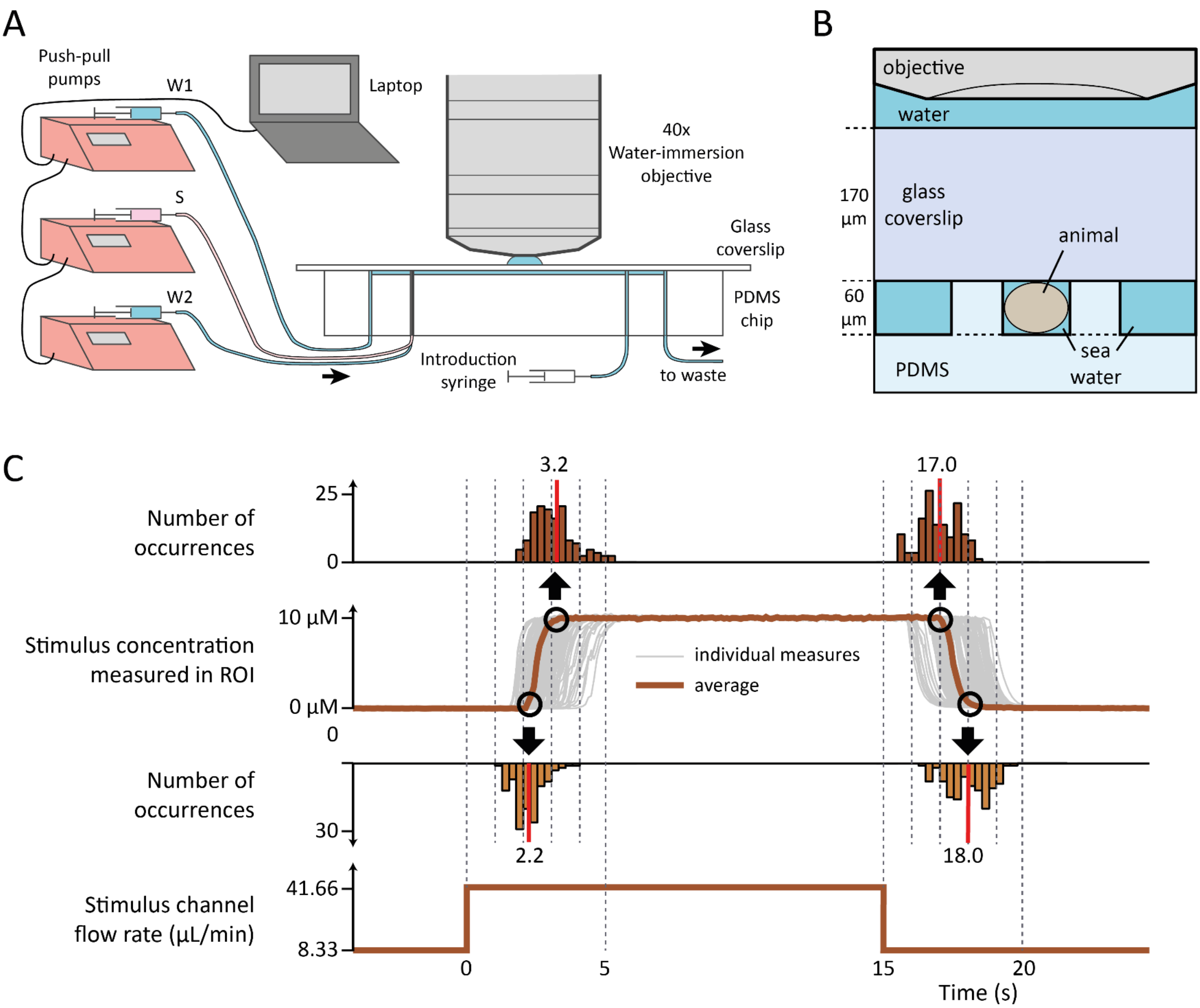

Figure S1

Additional descriptions of the experimental setup. (**A**) General schematic showing computer-controlled pumps, tubings and position of the device under the objective. (**B**) Detail of the mounting: cross-section through the microfluidic trap, illustrating the chamber's height compared to the coverslip thickness. (**C**) Quantification of stimulant concentration over time, as estimated by transmitted light intensity, measured in a square area located immediately upstream of the animal's head (see Figure 1G). Multiple measurements are shown as grey curves and their average as a thick brown curve. Distributions of the times of occurrence are shown for the four edges of the experimental curves (black circles), which correspond to beginning and end of stimulus onsets and offsets.

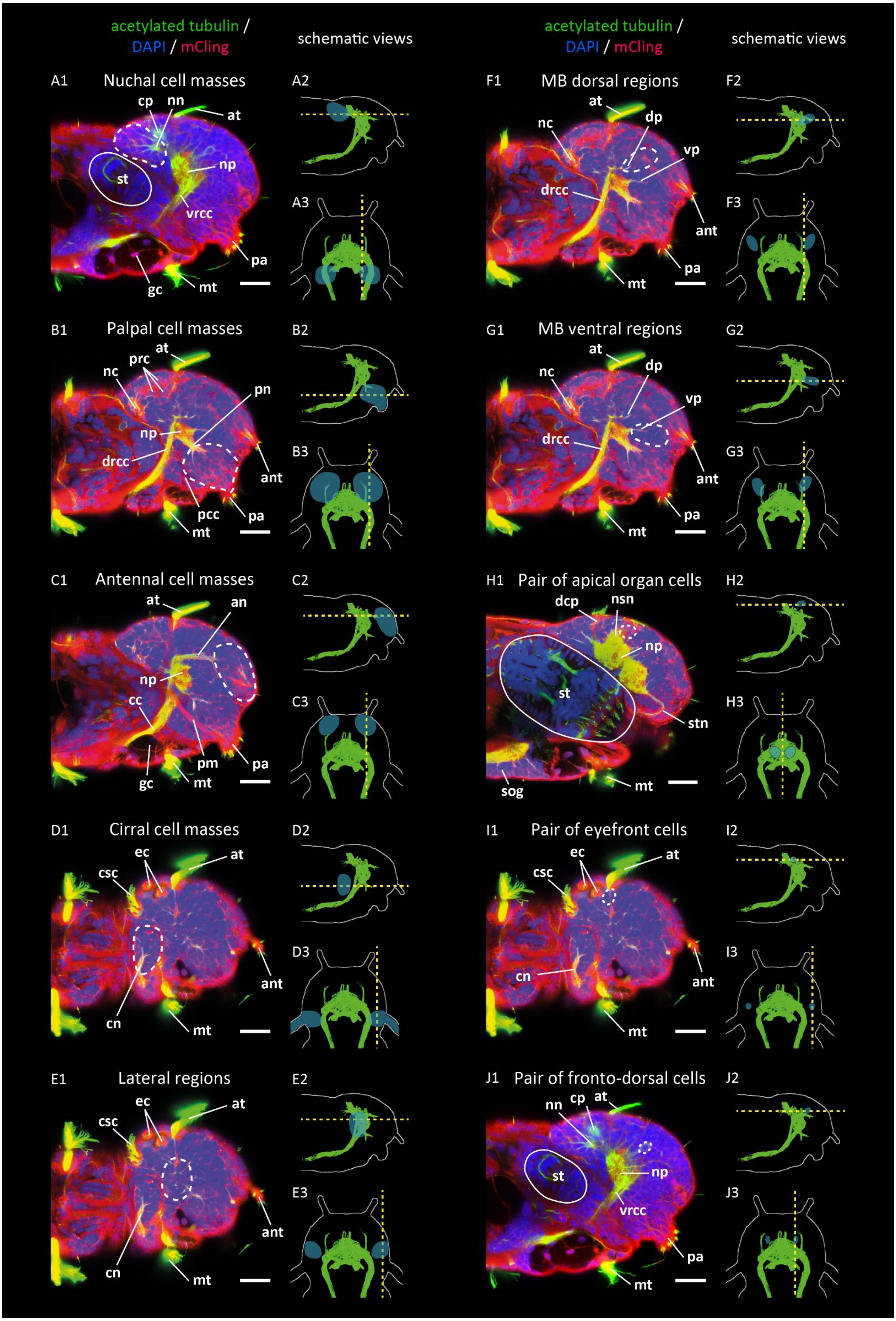
acetylated tubulin / DAPI / mCling
schematic views
acetylated tubulin / DAPI / mCling
schematic views
A1 Nuchal cell masses
cp nn at np st vrcc pa gc mt
A2
A3
F1 MB dorsal regions
at nc dp vp drcc ant pa mt
F2
F3
B1 Palpal cell masses
at prc nc pn np drcc ant pcc pa mt
B2
B3
G1 MB ventral regions
at nc dp vp drcc ant pa mt
G2
G3
C1 Antennal cell masses
at an np cc pm pa gc mt
C2
C3
H1 Pair of apical organ cells
dcp nsn np st stn sog mt
H2
H3
D1 Cirral cell masses
ec at csc ant cn mt
D2
D3
I1 Pair of eyefront cells
ec at csc cn ant mt
I2
I3
E1 Lateral regions
ec at csc ant cn mt
E2
E3
J1 Pair of fronto-dorsal cells
nn cp at st np vrcc pa mt
J2
J3

Figure S2 (previous page)

Anatomical stainings in lateral view, showing the head regions active in the context of chemical stimulations. (**A1**, **B1**, … **J1**) Acetylated tubulin immunostaining (green), counterstained for nuclear DNA (DAPI, blue) and membranes (mCling, red), averaged over several consecutive confocal planes. The approximate boundaries of the active regions are indicated by white dashed lines, the outlines of the stomodeum by white solid lines. an: antennal nerve; ant: antenna; cn: cirral nerve; dp: dorsal peduncle of the mushroom bodies; nc: nuchal cavity; nn: nuchal nerve; np: main neuropil; nsn: neurosecretory neuropil; pa: palp; pn: palpal nerve; prc: photo-receptor cells; vp: ventral peduncle of the mushroom bodies. Further abbreviations: see abbreviations list. Scale bars: 20 µm. (**A2**, **B2**, … **J2**) Schematic representation of the head in lateral view, showing its outlines (grey), a thresholded maximum projection of the nervous fibres (green) and the area containing the cell bodies of the corresponding region (shaded blue). The position of the horizontal plane shown in the corresponding images in Figure 2 is indicated by a yellow dashed line. (**A3**, **B3**, … **J3**) Same in dorsal view. The position of the sagittal plane shown in the adjacent anatomical staining is indicated by a yellow dashed line.

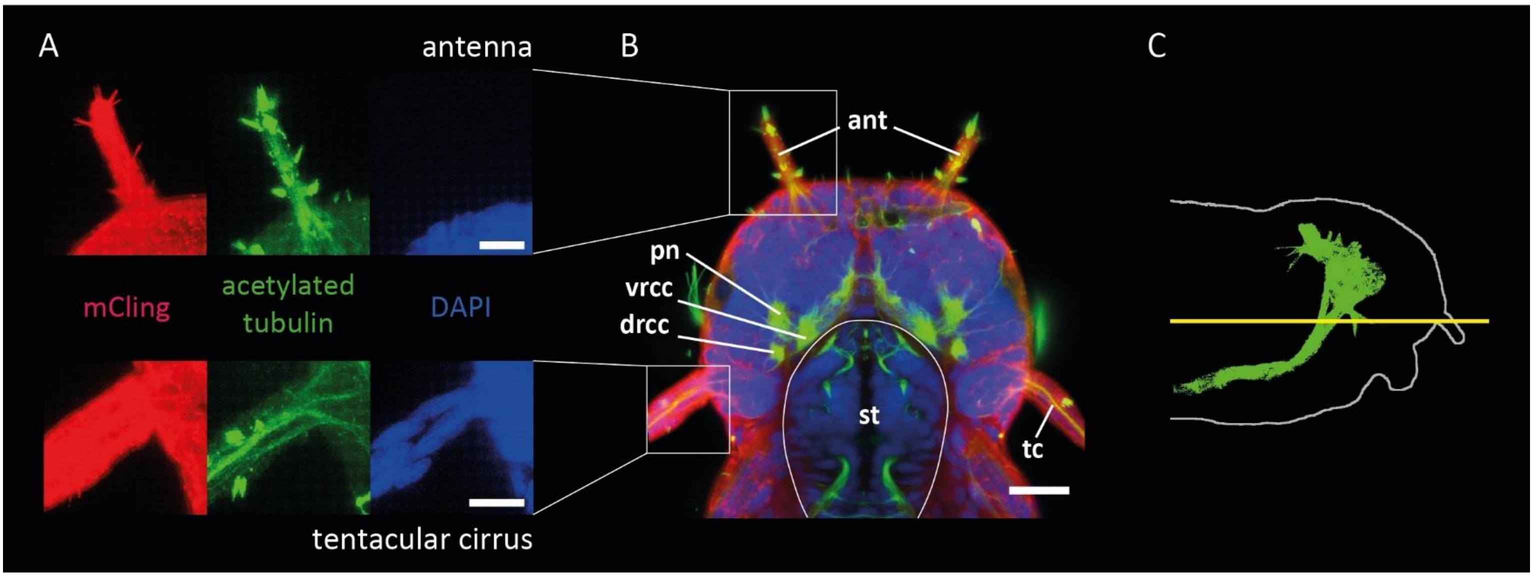

Figure S3

Anatomical details of the antennae and tentacular cirri at 6dpf. (**A**) Close-up of head appendages, showing that cell bodies (DAPI nuclear DNA staining, blue) are present in the appendage of the tentacular cirrus (bottom), but not of the antenna (top). Both appendages contain membrane extensions (mCling membrane staining, red), nervous fibres and clusters of sensory cilia (acetylated tubulin immunostaining, green). (**B**) Head overview in a horizontal plane. The outlines of the stomodeum are indicated by white solid lines. ant: antennae; pn: palpal nerve; st: stomodeum; tc: tentacular cirrus. Further abbreviations: see abbreviations list. (**C**) Schematic representation of the head in lateral view, showing its outline (grey), a maximum projection of the nervous fibres (green). The position of the horizontal plane shown in (**B**) is indicated by a yellow solid line. Scale bars: 10 µm in (**A**), 20 µm in (**B**).

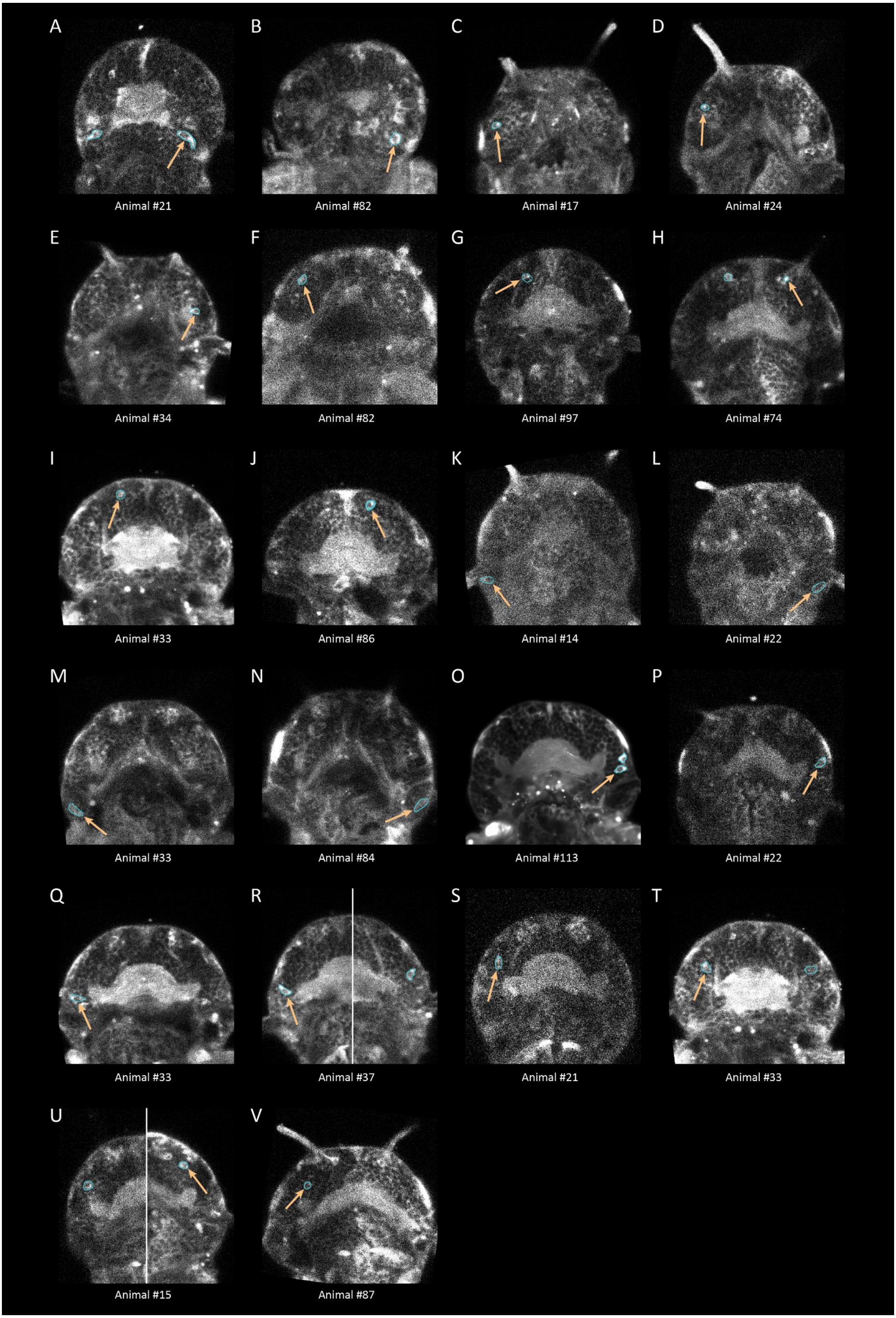
A
Animal #21
B
Animal #82
C
Animal #17
D
Animal #24
E
Animal #34
F
Animal #82
G
Animal #97
H
Animal #74
I
Animal #33
J
Animal #86
K
Animal #14
L
Animal #22
M
Animal #33
N
Animal #84
O
Animal #113
P
Animal #22
Q
Animal #33
R
Animal #37
S
Animal #21
T
Animal #33
U
Animal #15
V
Animal #87

Figure S4 (previous page)

Calcium snapshots and regions of interest used to generate the calcium traces shown in Figure 3C. GCaMP6s signal in single confocal planes, averaged over several consecutive time points. Individual responsive cell bodies are circled in cyan; the orange arrows point at the cells whose trace is shown in Figure 3C. (**A-B**) nuchal cell masses, (**C-F**) palpal cell masses, (**G-J**) antennal cell masses, (**K-N**) cirral cell masses, (**O-R**) lateral regions, (**S-T**) MB dorsal regions, (**U-V**) MB ventral regions.

Figure S5 (next page)

Calcium snapshots and regions of interest used to generate the calcium traces shown in Figure 5. GCaMP6s signal in single confocal planes, averaged over several consecutive time points. Individual cell bodies or neuropils whose trace are shown in Figure 5A or 5C are indicated by an orange arrow. The insets in (**E**) and (**H**) are taken from a neighbouring confocal plane. (**A-H**) apical organ cells and neurosecretory neuropil (nsn), (**I-K**) eyefront cells, (**L-Q**) fronto-dorsal cells.

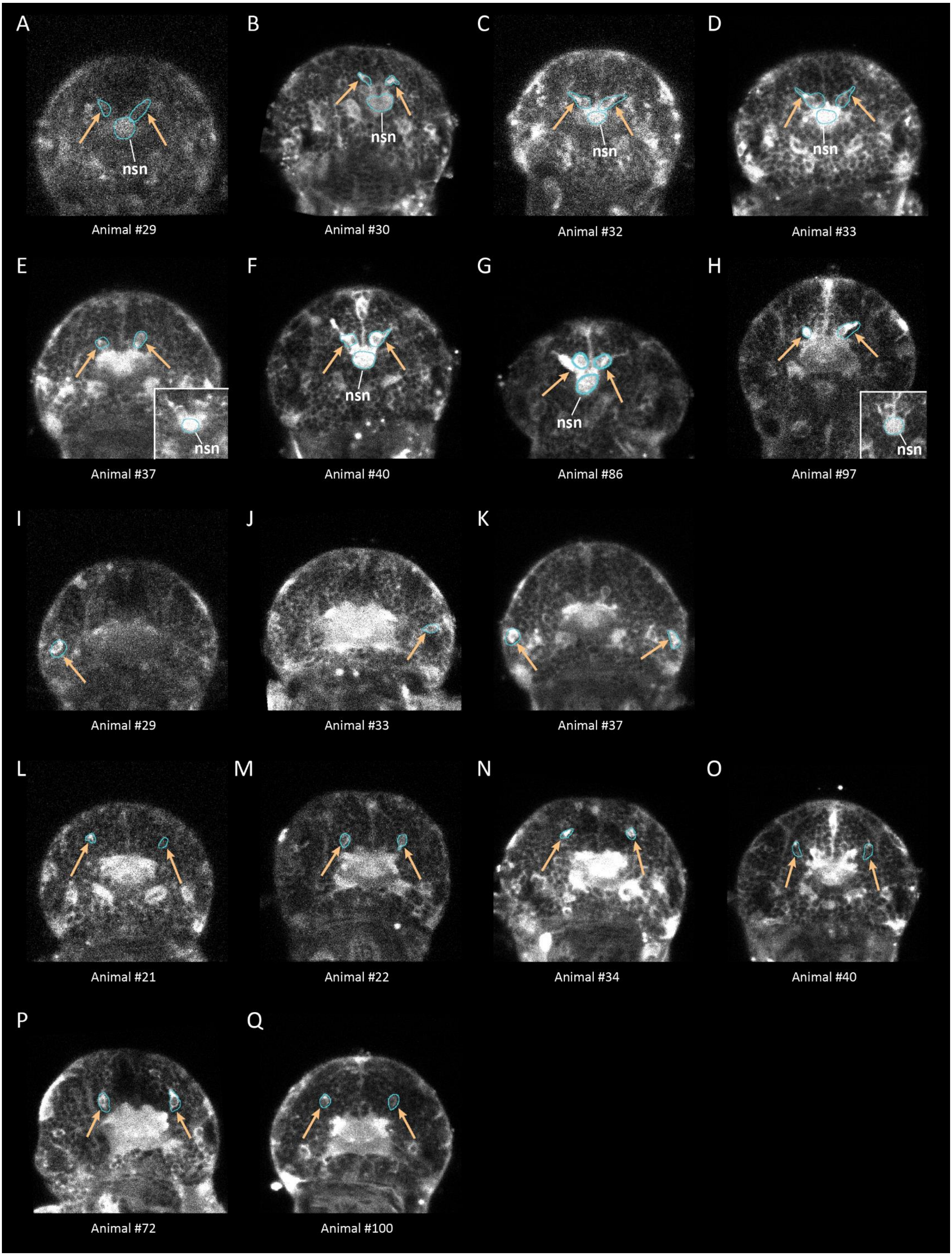
A
nsn
Animal #29
B
nsn
Animal #30
C
nsn
Animal #32
D
nsn
Animal #33
E
nsn
Animal #37
F
nsn
Animal #40
G
nsn
Animal #86
H
nsn
Animal #97
I
Animal #29
J
Animal #33
K
Animal #37
L
Animal #21
M
Animal #22
N
Animal #34
O
Animal #40
P
Animal #72
Q
Animal #100

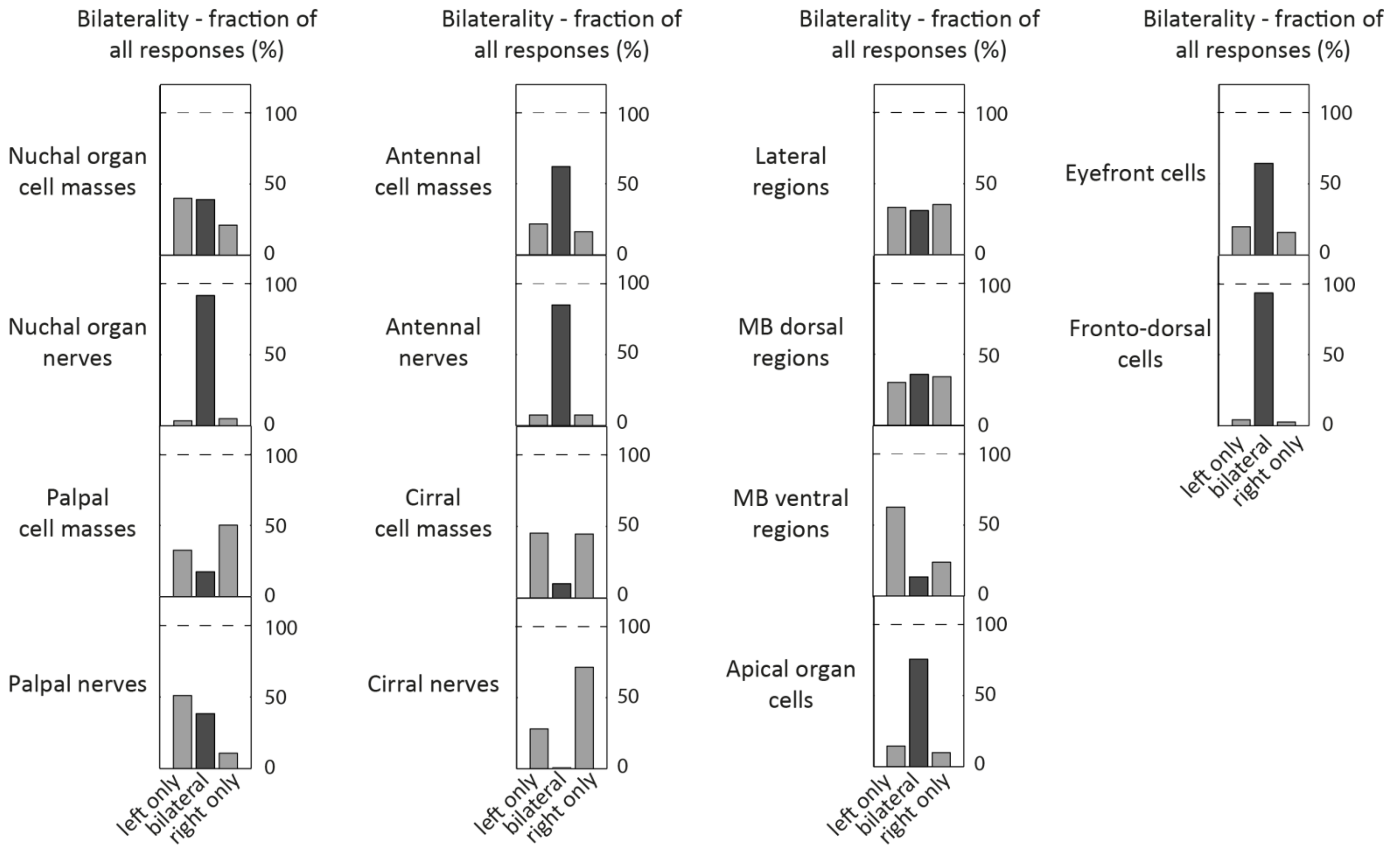

Figure S6

Quantification of response bilaterality for each region.

	groups compared		difference between group means	p-value	95% confidence interval of the true difference of means	
					lower limit	upper limit
Nuchal organs	1-butanol	amyl acetate	-1,6488	0,9984	-29,0253	25,7277
	1-butanol	glutamate	9,1537	0,801	-18,2228	36,5302
	1-butanol	sucrose	-8,2625	0,845	-35,6391	19,114
	amyl acetate	glutamate	10,8025	0,6898	-15,7567	37,3616
	amyl acetate	sucrose	-6,6138	0,9054	-33,1729	19,9454
	glutamate	sucrose	-17,4162	0,302	-43,9754	9,1429
Antennae	1-butanol	amyl acetate	11,4198	0,7255	-18,2804	41,1199
	1-butanol	glutamate	9,9868	0,7983	-19,7133	39,6869
	1-butanol	sucrose	14,3519	0,5628	-15,3483	44,052
	amyl acetate	glutamate	-1,433	0,9991	-30,2463	27,3804
	amyl acetate	sucrose	2,9321	0,9925	-25,8813	31,7454
	glutamate	sucrose	4,3651	0,9761	-24,4483	33,1784
Palps	1-butanol	amyl acetate	-9,2959	0,9321	-51,5404	32,9485
	1-butanol	glutamate	-37,788	0,0926	-80,0324	4,4564
	1-butanol	sucrose	-15,0279	0,7698	-57,2723	27,2166
	amyl acetate	glutamate	-28,4921	0,2543	-69,4752	12,4911
	amyl acetate	sucrose	-5,7319	0,981	-46,715	35,2512
	glutamate	sucrose	22,7601	0,4455	-18,223	63,7433
Tentacular cirri	1-butanol	amyl acetate	9,9916	0,895	-28,5666	48,5497
	1-butanol	glutamate	-31,6707	0,1377	-70,2288	6,8875
	1-butanol	sucrose	-10,5331	0,8796	-49,0913	28,025
	amyl acetate	glutamate	-41,6623	0,0244	-79,0692	-4,2554
	amyl acetate	sucrose	-20,5247	0,456	-57,9316	16,8822
	glutamate	sucrose	21,1376	0,4304	-16,2693	58,5445

(peach)	p-value > 0.05
(green)	p-value < 0.05

Table S1

Multiple comparison test based on one-way ANOVA, for differential responses to stimulants.
